# The role of auditory and haptic cues in object enumeration within containers

**DOI:** 10.1007/s00221-025-07215-4

**Published:** 2026-01-23

**Authors:** Ilja Frissen, Olga Sagou, Krista E. Overvliet

**Affiliations:** 1 School of Information Studies, McGill University, Montreal, Canada; 2 Experimental Psychology and Helmholtz Institute, Utrecht University, Utrecht, The Netherlands

**Keywords:** Numerosity, Haptics, Auditory, Multisensory, Human

## Abstract

Everyday experience suggests that handheld containers convey auditory and haptic information about their contents. The primary objective of this study was to examine how these two sources of information interact. Across three experiments, participants estimated the number of beads in cardboard boxes under three sensory conditions: haptics-only, auditory-only, and auditory-haptic. We hypothesized that the auditory-haptic condition would yield more accurate and precise estimates. A secondary objective was to assess the effects of time and manipulation constraints. In Experiment 1, participants were limited to 5 s of exploration using a standardized movement; Experiment 2 removed the time constraint, and Experiment 3 additionally removed the movement constraint. Results indicated that participants could reliably estimate the number of items under all conditions but provided little evidence for multisensory integration. When both constraints were removed, the haptic-only condition produced significantly more accurate estimates. These findings suggest that manipulation constraints influence enumeration performance and that haptic cues can support accurate judgments when exploration is unconstrained.

## Introduction

Humans have long relied on containers for storage and transport (Bevan 2014; Klose 2015), but little is known about how we infer their contents from sensory cues. Containers are likely to have been part of the human experience for as long as humans have been around (Bevan 2014; Suddendorf and Langley, 2020). They are thought to have been instrumental in hominin evolution (e.g., Suddendorf et al. 2020) and have long since been cultural universals (Goodenough 2003).

One general question that arises from the long common history of humans and containers is whether we can make reliable perceptual inferences about a container’s content. Daily experiences imply that we can: Shaking a milk carton or a box of chocolate sprinkles allows for clear impressions of how much content there is. As in this example, most common interactions with a container produce both auditory and haptic cues. Here we investigate how effective either of these two cues are, as well as how they interact in making inferences about the number of objects in a container (i.e., enumeration).

### Background

Besides the daily experiences, prior research shows that auditory and haptic cues can each support judgments about container contents, yet their combined effect remains unclear. Auditory cues have been shown to afford inferences about the fullness of a vessel from the sound of poured liquids (Cabe and Pittinger, 2000), about whether contained water was hot or cold (Velasco et al. 2013), and how many objects are inside a box (i.e., enumeration; Hummel et al. 2022; Pittenger et al. 1997; Pittenger and Mincy 1999; Plaisier and Smeets 2017). Haptic cues afford accurate judgments about how many deciliters there are in a milk carton (Jansson 1993; Jansson et al. 2006) or milliliters in a bottle (Koshiyama et al. 2015), and tracking moving objects inside tubes (Frissen et al. 2022; Yao and Hayward 2006). There are also several haptic enumeration studies (Frissen and Chen 2024; Frissen et al. 2023, 2025; Hummel et al. 2022; Sekiguchi et al. 2005). For instance, Frissen et al. (2023) reported on a series of three experiments in which participants handled small cardboard jewelry boxes holding between one and five beads of various diameters and weights and made direct estimates of the number of objects. Overall, this, and similar enumeration studies document remarkably accurate performance although with a progressive tendency to underestimate as the number of objects increases.

Early work on combined auditory and haptic cues was conducted by Pittenger and colleagues (1997; Pittenger and Mincy 1999). These studies examined whether participants could reliably rank a series of containers based on the perceived size of balls inside of the containers. The containers were explored in one of two ways. In one condition, the containers (opaque plastic cylinders) were shaken and in a second condition, the content of containers (glass bowls) was stirred with a stick. Access to sensory information was controlled by who was allowed to interact with the container. For the haptic-only condition, participants handled the containers while sounds were masked. For the auditory-only condition, the experimenter handled the container while the participant listened. And for the haptic-auditory condition the participant handled the container without the sounds being masked. Participants performed above chance with either cue, but combining cues offered no advantage over auditory information alone. Similarly, Hummel et al. (2022) found that auditory cues yielded slightly more accurate enumeration than haptic cues, and that combined cues were comparable to auditory-only performance. Together, these findings suggest that haptic information adds little to the multisensory percept, implying a winner-take-all process that favors auditory cues.

One study, however, challenges this interpretation. Plaisier and Smeets (2017) asked participants to estimate the number of wooden spheres (one to five) in small cardboard boxes after five seconds of handling. The task included two conditions: in one, participants had access to both auditory and haptic cues; in the other, they heard audio recordings from the first condition. Results showed that the auditory–haptic condition produced significantly more accurate and less variable estimates than the auditory-only condition. The authors argued that this improvement reflects a multisensory integration process, whereby combining cues reduces sensory noise and enhances perceptual precision (Faisal et al. 2008; Ernst and Bülthoff 2004; Stein, 2012).

However, the previous auditory–haptic studies have notable limitations. They rely exclusively on group-level analyses, overlooking individual differences, and most are based on very small samples (e.g., Plaisier and Smeets 2017) or presented only as brief summary reports (Hummel et al. 2022; Pittenger et al. 1997; Pittenger and Mincy 1999). These limitations have two important implications. First, the absence of individual-level analyses undermines any conclusion that auditory cues are the sole “winner” in a winner-take-all mechanism (Pittenger et al. 1997; Pittenger and Mincy 1999). It is plausible that some participants relied more on haptic cues, but such patterns were obscured in group averages. Second, the small sample size in Plaisier and Smeets (2017)—only seven participants—and reliance on group-level variability estimates limit the reliability of their precision measure and, by extension, their claim of sensory integration.

### The present study

The primary objective of the present study is to investigate the role of auditory and haptic cues in the estimation of the number of objects inside handheld boxes. We take an analytical approach that can show individual changes in precision (Scheller and Nardini 2024) as well as suggest individual “winners” for auditory or haptic cues. We adopt *integration* as the default position and hypothesize that the estimations of the number of objects in a container with both auditory and haptic information are more accurate (i.e., responses being closer to the actual number of objects) and more precise (i.e., having a lower variability in responses) in comparison to having only auditory or haptic information.

A secondary objective pertains to the effects of how participants manipulate the box. To date, enumeration studies with handheld boxes have constrained *how long* participants are allowed to explore a box (typically between 5 and 10 s) while at the same time they have not controlled *how* participants are allowed to manipulate the box. Seminal studies on form recognition show that the haptic system performs exceptionally well under ecologically valid conditions (Gibson 1962; Heller 1984). Gibson (1962) reported near-perfect recognition (95%) when participants actively explored objects compared to passive presentation (49%). Similarly, Heller (1984) found that allowing 30 s of exploration yielded better performance (95%) than 5 s (71%). Here we increasingly reduce the amount of constraint on how participants could explore the boxes across a series of three experiments. In Experiment 1 participants were instructed to manipulate the boxes for exactly 5 s using a standardized manipulation method. In Experiment 2 participants still used the standardized manipulation method but were given no time constraint. In Experiment 3 participants were free to manipulate the boxes as they wished without time constraints.

## Experiment 1: Time limited, standardized movement

### Method

#### Participants

Eighteen participants within the age range of 22–59 years took part in the study (8 females; 1 left-handed). All participants reported normal hearing and haptic perception. Informed consent was obtained from all participants before their participation. The study protocol received approval from the Utrecht University Faculty of Social Sciences ethics committee. Participants participated voluntarily and were paid 8 euros per hour or received course credits. No power analysis was conducted; sample sizes were based on previous work on container haptics showing robust findings at the individual level with sample sizes between 15 and 24 (Frissen and Chen 2024; Frissen et al. 2023, 2025).

#### Stimuli and apparatus

**Fig. 1:**
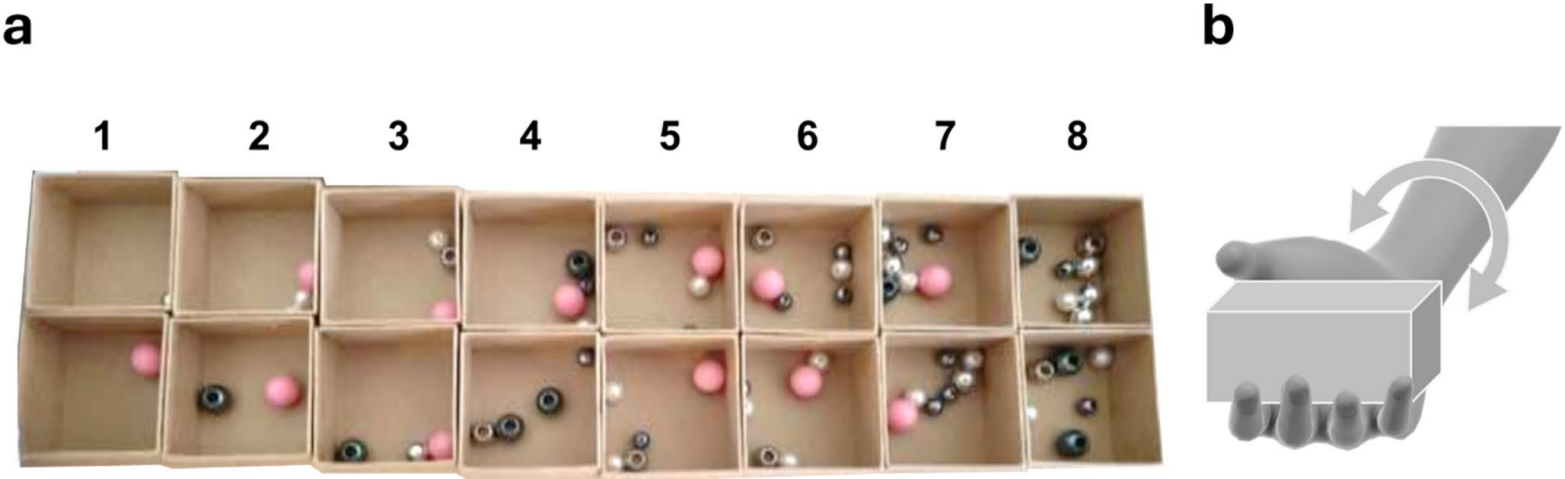
**a** Beads distribution inside the boxes with open lid. **b** Participants in Experiment 1 and 2 were instructed to move the box back and forth by rotating at the wrist (see also text)

One to eight beads of were distributed among two sets of eight cardboard boxes, each weighting 17.5 g plus a 9.2 g lid, and measuring 7.5 (L) × 7.5 (W) × 4.5 cm (H), for a total of 16 boxes (see Fig. 1a and Table 1 for details). There were two different boxes of 1 bead, two different boxes of 2 beads, and so on. All boxes had lids so that the content was hidden from view. While previous studies have suggested that weight is only a minor cue in determining the number of objects in a box (Frissen et al. 2023, 2025), care was taken to minimize the weight differences between the boxes by using beads of different weights, sizes, and materials, resulting in weights ranging between 28.3 and 35.2 g. To aid the researcher, stickers were added at the bottom of the boxes indicating the number of items.

**Table 1:** Distribution of objects and weights of items in the experimental containers

#Objects (total)	#Objects (per type)						Weight (g)	
	Pink	Blue	Silver1	Silver2	Pearl	Gray	Beads	Total
	(3.2 g)	(1.6 g)	(1.4 g)	(0.9 g)	(0.8 g)	(0.4 g)		
1	0 | 1	1 | 0	0 | 0	0 | 0	0 | 0	0 | 0	1.6 | 3.2	28.3 | 29.9
2	1 | 1	0 | 0	0 | 1	1 | 0	0 | 0	0 | 0	4.1 | 4.6	30.8 | 31.3
3	1 | 1	0 | 0	0 | 1	1 | 0	1 | 0	0 | 1	4.9 | 5.0	31.6 | 31.7
4	1 | 0	0 | 2	0 | 1	0 | 1	2 | 0	1 | 0	5.2 | 5.5	31.9 | 32.2
5	1 | 1	0 | 0	0 | 0	1 | 1	1 | 2	2 | 1	5.7 | 6.1	32.4 | 32.8
6	1 | 1	0 | 0	1 | 0	1 | 1	1 | 3	2 | 1	7.1 | 6.9	33.8 | 33.6
7	0 | 1	2 | 0	2 | 1	0 | 0	1 | 2	2 | 3	7.6 | 7.4	34.3 | 34.1
8	0 | 1	1 | 1	3 | 0	1 | 1	1 | 2	2 | 3	8.3 | 8.5	35.0 | 35.2

Information for the two sets of containers (see text) is separated by a pipe symbol (i.e., Set 1 | Set 2)

The randomization of trials, time keeping, and recording of answers, were controlled by a custom routine implemented in PsychoPy (version 2022.2.5), a software package for creating psychological experiments (Peirce et al. 2019). Four versions of the experiment were developed: a practice round and three experimental conditions.

#### Design and procedure

The experiment followed a 3 (Modality: Auditory-Only [A], Haptic-only [H], Auditory-Haptic [AH]) × 8 (Number of Objects: 1–8) × 4 (Repetitions) within-participants design for a total of 96 trials per participant. The levels of the Modality factor were counterbalanced, and Number of Objects was presented in a random order (in which each copy of a box was repeated twice). The experimental session lasted about 45 min.

The participant was seated at a desk next to the experimenter, so that the experimenter could easily hand over the boxes. A computer screen with trial information separated the experimenter from the participant, so that the participant could not see the labels on the boxes.

In the H and AH conditions, the participants manipulated the boxes themselves. In the A condition the experimenter manipulated the box in the standardized way (as described below) near the participant. In the haptic-only condition, participants wore noise-cancelling headphones (Bose NC700) playing white noise to block out any auditory information. Participants were not blind-folded.

In each trial the experimenter placed the box with the lid facing up in the participant’s hand (or the experimenter’s hand for the A condition). Subsequently, a beep noise was played to indicate to the participant (or the experimenter in the auditory condition) that they could start manipulating the box. After 5 s, another beep indicated the end of the manipulation time and the participant stated their numerosity estimation, which was recorded by the researcher on the computer. The box was returned, and the next trial began. There was neither mention of the maximum number of items to the participants, nor feedback on performance.

How participants manipulated the box was standardized based on a pilot study. Eight participants were instructed to manipulate, as they wished and without time limit, three different boxes containing 3, 5, or 8 items and to estimate the number of items inside the box. A number of manipulation strategies were observed, such as, shaking the box vigorously and moving the box horizontally in circles. However, the most common method involved carefully moving the box back and forth by rotating at the wrist (See Fig. 1b). It was therefore decided to instruct all participants in the experiment to use this particular manipulation technique. Before data collection started, participants were given a practice round to familiarize themselves with the task and the experimental setup. This consisted of three randomly selected trials from the AH condition.

#### Dependent variables and data analysis

We considered three dependent measures: 1) the estimated number of objects, and 2) how accurate and 3) precise those estimates are. Number estimates were calculated for each level of Number of Objects (1 – 8) by simply taking the mean across the four Repetitions. Both accuracy and precision were calculated for each participant individually using all 32 (8 × 4) number estimations collected for each of the three Modality conditions. Accuracy was operationalized in terms of the root-mean-square of errors (RMSE) in number estimations. Precision was operationalized in terms of the RMSE of the residuals from a second-order polynomial regression.^1^ Scheller and Nardini (2024) have pointed out that, in the multisensory perception literature, the most common approach to studying integration has been to compare precision when two senses are combined with precision when the two senses are on their own. That is, noise_1+2_ vs. noise_1_ and noise_1+2_ vs. noise_2_. However, they also demonstrate, using computational simulations, that this common approach is sensitive to high rates of false positives (concluding there was integration, when there was none). Here we therefore adopted their “individually-determined best cue analysis”, in which, for each participant it was determined which of the two senses produced the more precise (e.g., least noisy) estimates, which then served as the comparator; that is, noise_1+2_ vs. min(noise_1_, noise_2_).

Data processing and visualization were done with Matlab (The MathWorks Inc., 2022). Inferential statistical analyses, assuming a level of significance of 5%, were performed in JASP (JASP Team 2023). For repeated measures ANOVAs, violations of the assumption of sphericity were addressed using the Greenhouse–Geisser correction (in which case we report non-integer values for corrected degrees of freedom). All post-hoc tests were Holm-corrected for multiple comparison. For effect sizes, we report Generalized Eta-Squared values (η^2^_G_) for ANOVAs (Bakeman 2005) and Cohen’s d for any pairwise comparisons.

### Results and discussion

The results show that with a time limit and a standardized movement, there is little evidence to support the idea that participants determined their estimates in the AH condition by integrating auditory and haptic information.

#### Number estimates

**Fig. 2:**
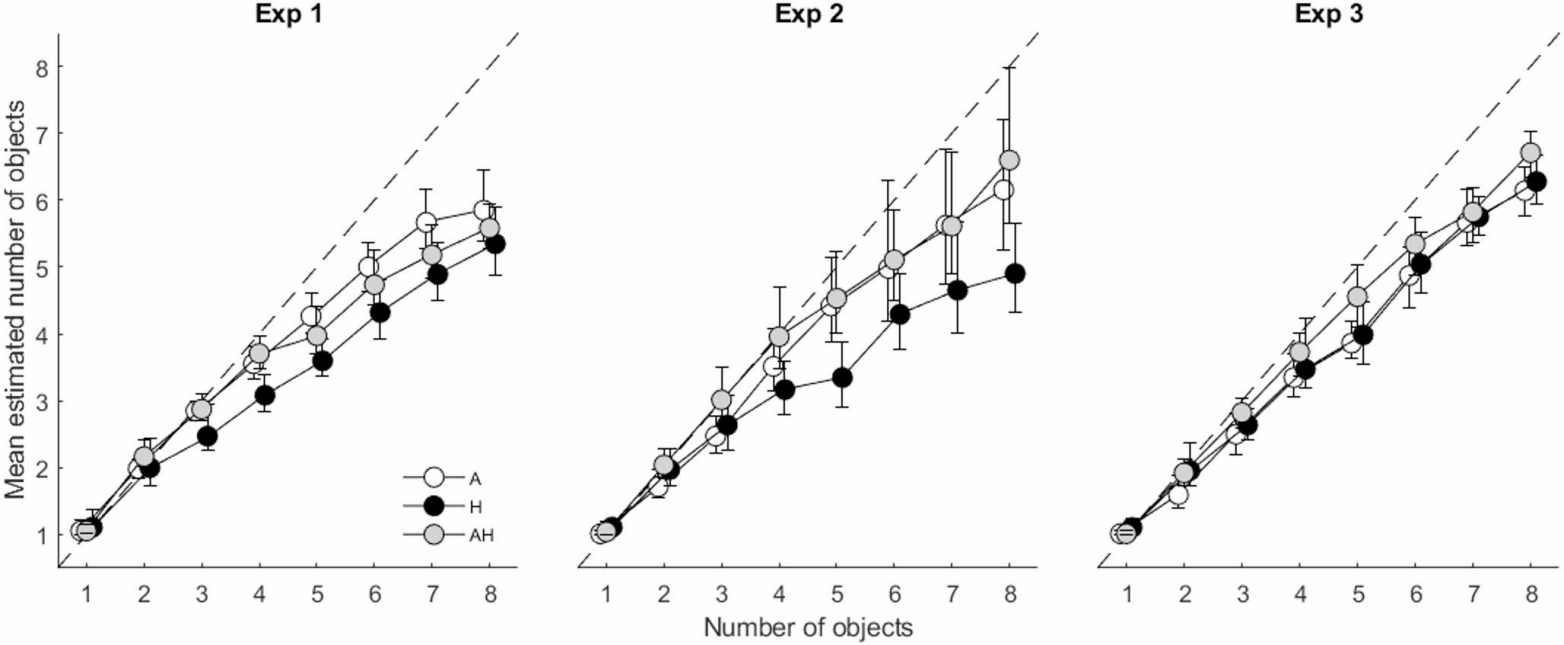
Mean estimated number of objects for Experiments 1–3. Error bars represent 95% empirical bootstrapped (n = 10,000) confidence intervals using the bias corrected and accelerated percentile method (Haukoos and Lewis 2005)

Figure 2a plots the group average estimates for Experiment 1, which allows two observations. The first observation is that, among the three conditions, A tended to produce the most accurate estimates on average, followed by AH, and then by H. This observation was supported by a 3 (Condition: A, H, AH) × 8 (Number of Objects: 1—8) repeated measures ANOVA (see Table 2 for details) that showed that the main effects of Condition and Number of Objects were significant. However, whereas the effect size for Number of Objects was, unsurprisingly, very large (η^2^_G_ = 0.817), for Condition it could be considered medium (η^2^_G_ = 0.062). The main effect of Condition was further explored with post-hoc comparisons, which showed significant differences for A vs. H and for H vs. AH, but not for A vs. AH. The interaction between Condition and Number of Objects was significant as well although its effect size was small to medium (η^2^_G_ = 0.037). The interaction could be attributed to the fact that the differences between the three conditions tended to increase as the number of objects in the box increased.

**Table 2:** Number estimates

	ANOVA							Post-hoc tests			
Exp	Effect	df*	F	MSE	*p*	η2_G_		Mean Difference [95% CI]	Cohen’s *d* [95% CI]	*t*	*d*
1	Condition	2, 34	9.12	6.94	< 0.001	0.062	A-H	0.43 [ 0.17; 0.68]	0.59 [ 0.15; 1.04]	4.14	< 0.001
	number of objects	1.8, 30.5	250.11	527.64	< 0.001	0.817	A-AH	0.12 [− 0.14; 0.37]	0.16 [− 0.21; 0.53]	1.15	0.259
	interaction	5.6, 94.9	2.97	1.47	0.013	0.037	H-AH	− 0.31 [ 0.57; − 0.05]	− 0.43 [− 0.83; − 0.02]	2.99	0.010
2	Condition	1.3, 22.2	9.2	30.13	0.004	0.049	A-H	0.48 [ 0.04; 0.91]	0.35 [− 0.01; 0.71]	2.76	0.018
	number of objects	1.4, 23.0	89.68	793.03	< 0.001	0.583	A-AH	− 0.25 [− 0.69; 0.18]	− 0.18 [− 0.52; 0.15]	1.46	0.153
	interaction	4.1, 70.1	5.94	7.65	< 0.001	0.040	H-AH	− 0.73 [− 1.16; 0.29]	− 0.53 [− 0.93; − 0.13]	4.22	< 0.001
3	Condition	2, 34	3.58	4.81	0.039	0.040	A-H	− 0.15 [− 0.50; 0.19]	0.21 [− 0.67; 0.26]	1.13	0.268
	number of objects	7, 119	479.94	193.36	< 0.001	0.856	A-AH	− 0.21 [− 0.55; 0.13]	− 0.28 [− 0.75; 0.19]	1.54	0.267
	interaction	6.2, 103.1	1.14	0.73	0.35	0.020	H-AH	− 0.36 [− 0.71; 0.02]	− 0.49 [− 0.99; − 0.02]	2.67	0.035

Details for the repeated measures ANOVAs and post-hoc comparisons for the main effect of Condition

*non-integer values indicate Greenhouse–Geisser corrected degrees of freedom

The second observation is that the estimates for the AH condition were not better (i.e., more accurate) than those of the uni-modal conditions. In fact, the estimates for the AH condition tended to lie *between* those of A and H, which at face value could be taken as indicative of a form of integration based on a (weighted) average of the two uni-modal cues. However, the results for the Accuracy and Precision, to which we turn next, are not consistent with such an interpretation, since neither measure shows convincing improvement in performance in the AH condition relative to A or H.

#### Accuracy

**Fig. 3:**
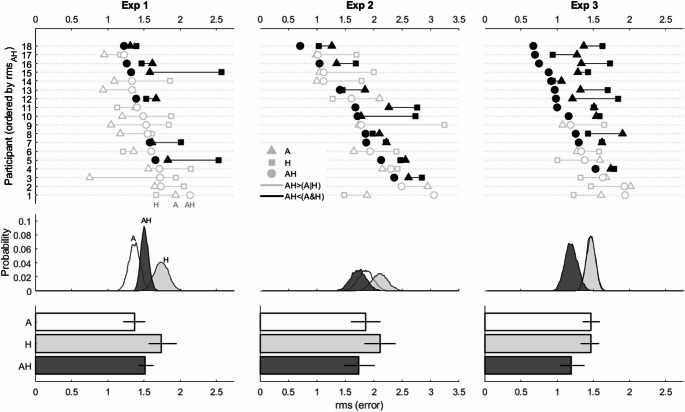
Tests for integration in terms of accuracy for Experiments 1–3. The top row panels show individual results; each line is one participant. The horizontal lines indicated RMS values ranging between the auditory-only (triangles) to haptic-only (squares) conditions. The circular marker shows RMS values when both cues were available. Only those markers to the left of the best condition are consistent with increased accuracy (shown in solid black). The middle row panels show the corresponding group level histograms based on empirical bootstraps of the individual mean RMS. The bottom row panels show the group level mean RMS values. Error bars represent 95% empirical bootstrapped (n = 10,000) confidence intervals using the bias corrected and accelerated percentile method (Haukoos and Lewis 2005)

The results for the accuracy analysis are shown in the left column panels of Fig. 3. At an individual level we saw that only 6 out of the 18 participants showed evidence of an increase in accuracy when both auditory and haptic cues were available (black markers). The majority of participants produced RMS values that were between the A and H conditions, and some produced RMS values that were even worse than the worst cues (e.g., participants 1 and 6). A 3 (Condition: A, H, AH) repeated measures ANOVA showed a significant main effect (F(2, 34) = 8.06, MSE = 0.636, *p* = 0.001, η^2^_G_ = 0.177). Post-hoc comparisons (see Table 3) showed significant differences for A vs. H and for H vs. AH, but not for A vs. AH.

**Table 3:** Accuracy and precision

	Accuracy				Precision			
Exp	Comp	Mean difference	*p*	Cohen’s *d*	Comp	Mean difference	*p*	Cohen’s *d*
1	A-H	− 0.37 [− 0.61; − 0.14]	< 0.001*	− 1.09 [− 1.92; − 0.26]	Best–Worst	− 0.20 [− 0.30; − 0.10]	< 0.001*	− 0.86 [− 1.45; − 0.28]
	A-AH	− 0.14 [− 0.38; 0.09]	0.136	− 0.42 [− 1.13; 0.29]	Worst-Both	0.10 [ 0.00; 0.20]	0.029*	0.44 [− 0.04; 0.92]
	H-AH	0.23 [− 0.01; 0.47]	0.039*	0.67 [− 0.07; 1.42]	Best-Both	− 0.10 [− 0.20; 0.00]	0.029*	− 0.43 [− 0.90; 0.05]
2	A-H	− 0.25 [− 0.59; 0.10]	0.155	− 0.42 [− 1.02; 0.19]	Best–Worst	− 0.25 [− 0.45; − 0.05]	0.012*	− 0.58 [− 1.12; − 0.04]
	A-AH	0.13 [− 0.21; 0.47]	0.352	0.22 [− 0.37; 0.80]	Worst-Both	0.16 [− 0.04; 0.36]	0.094	0.39 [− 0.12; 0.89]
	H-AH	0.38 [ 0.03; 0.72]	0.027*	0.63 [− 0.01; 1.27]	Best-Both	− 0.08 [− 0.28; 0.12]	0.312	− 0.19 [− 0.68; 0.29]
3	A-H	0.00 [− 0.23; 0.24]	0.977	0.01 [− 0.72; 0.74]	Best–Worst	− 0.18 [− 0.27; − 0.10]	< 0.001*	− 1.04 [− 1.71; − 0.37]
	A-AH	0.27 [ 0.04; 0.51]	0.017*	0.87 [ 0.05; 1.67]	Worst-Both	0.22 [ 0.13; 0.30]	< 0.001*	1.24 [ 0.51; 1.98]
	H-AH	0.27 [ 0.04; 0.50]	0.017*	0.86 [ 0.04; 1.68]	Best-Both	0.04 [− 0.05; 0.12]	0.308	0.20 [− 0.30; 0.71]

Details post-hoc comparisons. Values in square brackets are 95% CI

#### Precision

**Fig. 4:**
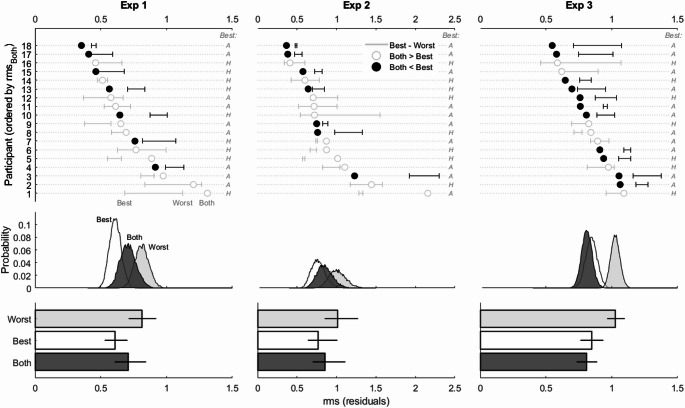
Tests for integration in terms of precision for Experiments 1–3. The top row panels show individual results; each line is one participant. The horizontal lines indicated RMS values for the best (leftward) and worst (rightward) cues. The circular marker shows RMS values when both cues were available. Only those markers to the left of the best cue are consistent with increased precision (shown in solid black). The labels on the right-hand side of each panel indicates whether the auditory (A) or haptic (H) condition produced the Best cue. The middle row panels show the corresponding group level histograms based on empirical bootstraps of the individual mean rms. The bottom row panels show the group level mean RMS values. Error bars represent 95% empirical bootstrapped (n = 10,000) confidence intervals using the bias corrected and accelerated percentile method (Haukoos and Lewis 2005)

The results for the precision analysis are shown in the left column panels of Fig. 4. At an individual level we saw that only 7 out of the 18 participants showed evidence of an increase in precision when both auditory and haptic cues were available (black markers). And of those 7 there were 2 that were only marginally better than the best cue (participants 15 and 17). The majority of participants produced RMS values that were between the best and worst cues, and some produced RMS values that were even worse than the worst cues (e.g., participants 1 and 5).

A 3 (Cues: Best, Worst, Both) repeated measures ANOVA showed a significant main effect (F(1.45, 24.79) = 12.82, MSE = 0.250, *p* < 0.001, η^2^_G_ = 0.116). More critically, the difference between the Best and Both cues conditions showed a decrease in precision when having both cues. However, while a post-hoc comparison suggested that the difference was statistically significant (*p* < 0.05) the corresponding confidence interval for Cohen d’s suggested that it was not so, since it included zero [− 0.90; 0.05])(See Table 3).

## Experiment 2: No time limit, standardized movements

### Method

Eighteen new participants within the age range of 19–44 years took part in the study (13 females; all right-handed). All participants reported normal hearing and haptic perception.

Experiment 2 had the same specifications as Experiment 1, except that there was no time limit on the (standardized) manipulation of the box. After the auditory starting signal, a timer started which was stopped when participants pressed a foot pedal to indicate that they were ready to give their numerosity estimation. Exploration times were saved to the computer alongside the numerosity estimation.

### Results and discussion

The results for Experiment 2 show that despite the removal of the time limit, performance is similar to Experiment 1. There was again little evidence for integration.

#### Number estimates

Figure 2 (middle panel) plots the group average estimates for Experiment 2, which allows for two main observations. First, the A and AH conditions now produced virtually identical estimates. Second, the H condition was (again) the least accurate of the three.

A 3 (Condition: A, H, AH) × 8 (Number of Objects: 1—8) repeated measures ANOVA showed that the main effects of Condition (F(1.30, 22.18) = 9.20, MSE = 30.13, *p* = 0.004; η^2^_G_ = 0.049) and Number of Objects (F(1.35, 22.99) = 89.68, MSE = 793.03, *p* < 0.001, η^2^_G_ = 0.58) were significant. Post-hoc comparisons for Condition showed significant differences for A vs. H (mean difference = 0.48, 95% CI = [0.04; 0.91], Cohen’s d = 0.35 [− 0.012; 0.71], t = 2.76, *p* = 0.018), and for H vs. AH (− 0.73 [− 1.16; 0.29], d = − 0.53 [− 0.93; − 0.13], t = 4.22, *p* < 0.001), but not for A vs. AH (− 0.25 [− 0.69; 0.18], d = − 0.18 [− 0.52; 0.15], t = 1.46, *p* = 0.15). The interaction between Condition and Number of Objects was significant as well (F(4.12, 70.08) = 5.94, MSE = 7.65, *p* < 0.001), with a small to medium effect size (η^2^_G_ = 0.040). Like in Experiment 1, the interaction could be attributed to the fact that some of differences among the three conditions tended to increase as the number of objects in the box increased as well.

#### Accuracy

The results for the accuracy analysis are shown in the center column panels of Fig. 3. At an individual level we saw that 8 out of the 18 participants showed evidence of an increase in accuracy when both auditory and haptic cues were available. The majority of participants produced RMS values that were between the A and H conditions, and some even produced RMS values that were even worse than the worst cues (e.g., participant 1). A 3 (Condition: A, H, AH) repeated measures ANOVA showed a significant effect (F(2, 34) = 4.95, MSE = 0.653, *p* = 0.029, η^2^_G_ = 0.067). Post-hoc comparisons showed significant differences for H vs. AH, but not for A vs. H and A vs. AH (Table 3).

#### Precision

The results for the precision analysis are shown in the center column panels of Fig. 4. They showed again that only 7 out of the 18 participants produced evidence for an increase in precision when both auditory and haptic cues were available and that the majority of participants produced RMS values that were between the best and worst cues, and several produced RMS values that were even worse than the worst cues. A 3 (Cues: Best, Worst, Both) repeated measures ANOVA showed a significant effect (F(1.47, 24.98) = 4.95, MSE = 0.381, *p* = 0.023, η^2^_G_ = 0.057). As in Experiment 1, the difference between the Best and Both cues showed a (non-significant) decrease in precision when having both cues and the confidence interval for Cohen d’s included zero [− 0.68; 0.29]) (See Table 3).

## Experiment 3: No time limit, no standardized movements

### Method

Eighteen new participants within the age range of 20–41 years took part in the study (10 females; 2 left-handed). All participants reported normal hearing and haptic perception.

Experiment 3 had the same specifications as Experiment 2, except that there was no restriction on the manipulation of the boxes; participants were free to move the box as they saw fit.

### Results and discussion

The results for Experiment 3 show that removing both the time limit and the movement constraint does show a benefit for the AH condition. However, the benefit is limited to accuracy and does not appear for precision. Moreover, we observed that H was now on par with A, suggesting that having a movement constraint might be part of the reason H had performed worst in the previous two experiments.

#### Number estimates

Figure 2 (right panel) plots the group average estimates for Experiment 3, which show that there were only minor apparent differences between the three conditions. That is, similar to Experiment 2, there was no apparent difference between the A and AH conditions. It was interesting, however, to see that the H condition now seemingly performed as well as the other two conditions, closing the gap that was observed in the previous two experiments.

A 3 (Condition: A, H, AH) × 8 (Number of Objects: 1—8) repeated measures ANOVA showed that the main effects of Condition (F(2,34) = 3.58, MSE = 4.81, *p* = 0.039; η^2^_G_ = 0.040) and Number of Objects (F(7, 119) = 479.94, MSE = 193.36, *p* < 0.001, η^2^_G_ = 0.86) were significant. Post-hoc comparisons for Condition showed a significant difference only for H vs. AH (mean difference = − 0.36, 95% CI = [− 0.71; 0.02], Cohen’s d = − 0.49 [− 0.99; − 0.02], t = 2.67, *p* = 0.035), But not for A vs. H (− 0.15 [− 0.50; 0.19], d = 0.21 [− 0.67; 0.26], t = 1.13, *p* = 0.268) or A vs. AH (− 0.21 [− 0.55; 0.13], d = − 0.28 [− 0.75; 0.19], t = 1.54, *p* = 0.27). Contrary to Experiments 1 and 2, the interaction between Condition and Number of Objects was not significant (F(6.24, 103.1) = 1.14, MSE = 0.73, *p* = 0.35) with a small effect size (η^2^_G_ = 0.020).

#### Accuracy

The results for the accuracy analysis are shown in the right column panels of Fig. 3. At an individual level we saw that 12 out of the 18 participants showed evidence of an increase in accuracy when both auditory and haptic cues were available, making up a majority. This time, only a minority of participants produced RMS values that were between the A and H conditions, and only one produced RMS values that were the worst (e.g., participant 1). A 3 (Condition: A, H, AH) repeated measures ANOVA showed a significant effect (F(1.47, 24.96) = 5.71, MSE = 0.602, *p* = 0.015, η^2^_G_ = 0.149). Post-hoc comparisons showed significant differences for A vs. AH and H vs. AH, but not for A vs. H (Table 3).

#### Precision

This time, the results for the precision analysis showed that the majority (11 out of the 18 participants) produced evidence of an increase in precision (Fig. 4). A 3 (Cues: Best, Worst, Both) repeated measures ANOVA showed a significant effect (F(2, 34) = 23.42, MSE = 0.243, *p* < 0.001, η^2^_G_ = 0.239). However, while the difference between the Best and Both cues did show an increase in precision for the latter, it was not statistically significant and the confidence interval for its corresponding Cohen d’s included zero [− 0.30; 0.71])(See Table 3).

## Across experiment comparison: effects of box manipulation constraints

A final set of analyses considered all three experiments together to assess how the gradually released box manipulation constraints affected number estimates, accuracy, and precision.

### Number estimates

A 3 (Experiment) × 3 (Condition: A, H, AH) × 8 (Number of Objects: 1—8) mixed ANOVA with Experiment as a between-subjects factor, showed that two of the three possible interactions involving the Experiment factor were significant; most important of which, the second-order interaction between all factors (F(15.26, 389.09) = 2.267, MSE = 1.20, *p* = 0.004; η^2^_G_ = 0.015).

This interaction was followed up by three separate 3 (Experiment) × 8 (Number of Objects) ANOVAs; one for each of the three levels of Condition. For the auditory-only condition there were no significant effects of Experiment (all F’s < 1). For the haptic-only condition there was a significant interaction (F(7.42, 189.30) = 3.52, MSE = 2.67, *p* = 0.001; η^2^_G_ = 0.055), which was attributed to the observation that estimates in Experiments 1 and 2 were virtually identical, while those in Experiment 3 were higher (i.e., more accurate) for larger numbers of objects (i.e., more than 5). For the auditory-haptic condition the interaction was not significant in the strict sense (F(7.42, 125.41) = 2.29, MSE = 3.29, *p* = 0.051); although it produced a small to medium effect size (η^2^_G_ = 0.037).

Thus, it could be argued that the second-order interaction in the omnibus analysis is attributable to the effect of the different box manipulation constraints across the three experiments. This effect was apparently absent for the auditory-only condition (where, contrary to the other conditions, the experimenter manipulated the box), marginal for the auditory-haptic condition, but pronounced for the haptic-only condition.

### Accuracy

A 3 (Experiment) × 3 (Condition: A, H, AH) mixed ANOVA with Experiment as a between-subjects factor, showed a significant main effect for Experiment (F(2, 51) = 10.90, MSE = 3.861, *p* < 0.001; η^2^_G_ = 0.210). This was attributed to the fact that RMS values were overall different between the experiments, being the largest for Experiment 2 (M = 1.90), intermediate for Experiment 1 (M = 1.54), and the smallest for Experiment 3 (M = 1.38). Post-hoc comparisons showed significant differences for Experiment 1 vs. Experiment 2 (*p* = 0.006) and for Experiment 2 vs. Experiment 3 (*p* < 0.001), but not for Experiment 1 vs. Experiment 3 (*p* = 0.156).

A significant main effect for Condition (F(1.66, 84.82) = 11.30, MSE = 1.457, *p* < 0.001; η^2^_G_ = 0.077) reflected the fact that H tended to produce less accurate responses compared to A and AH. Post-hoc comparisons showed significant differences for A vs. H (*p* = 0.003) and for H vs. AH (*p* < 0.001), but not for A vs. AH (*p* = 0.526). The interaction was not significant (F(6.33, 84.82) = 2.42, MSE = 0.312, *p* = 0.065; η^2^_G_ = 0.035).

### Precision

A 3 (Experiment) × 3 (Cues: Best, Worst, Both) mixed ANOVA with Experiment as a between-subjects factor, showed a significant effect for Cues only (F(1.61, 82.19) = 23.92, MSE = 0.647, *p* < 0.001; η^2^_G_ = 0.087), but not for any of the effects involving the Experiment factor (main effect: *p* = 0.079, η^2^_G_ = 0.077; interaction: p = 0.370, η^2^_G_ = 0.007). The (non-significant) main effect of Experiment could be attributed to the fact that RMS values for Experiment 1 were overall smaller than those for Experiments 2 and 3, which were comparable. Thus, there was no statistical evidence for an effect of the different box manipulation constraints on precision.

#### Individual best cues

The auditory cue was Best for 9/18 (50.0%), 10/18 (55.6%), and 11/18 (61.1%) participants, for the three experiments respectively, and 30/54 (55.6%) across all experiments. In other words, there does not appear to be a systematic advantage for either the auditory or the haptic condition. Instead, there seem to be individual differences in which sense allows for the more precise performance.

## General discussion

Our results show that participants could estimate the number of items in closed containers reasonably well, although they showed some general underestimation with higher numbers. The performance in terms of accuracy in the auditory-haptic condition and the auditory condition was quite similar, while in the haptic condition participants underestimated the number of items compared to the other two conditions. This difference became smaller when participants were completely free in their manipulation of the boxes (i.e., no time or strategy restrictions). When looking at performance in terms of precision, to investigate “winner-takes-all” or “integration” mechanisms, we found that this task yielded neither of these mechanisms: about half of the participants seemed to “favor” the auditory cue, while the other half “favored” the haptic cue.

The results of the auditory-only and haptic-only conditions were consistent with earlier work in that either of them was sufficient to make reasonably accurate inferences about the number of objects in a box. The results also reconfirmed a general underestimation of the number that increases with the number of items presented (Frissen et al. 2023; 2025; Hummel et al. 2022; Plaisier and Smeets 2017; Pittenger et al. 1997; Pittinger and Mincy, 1999).

### Little evidence for integration

However, our results are not consistent with the assertions put forward in earlier studies concerning how auditory and haptic information interact when both are available. First, our hypothesis was that auditory and haptic information would be integrated and that the estimations of the number of objects in a container with both auditory and haptic information would be more *accurate* and more *precise* in comparison to having only auditory or haptic information. Overall, we did not find any evidence in favor of this hypothesis, as we observed neither better accuracy nor better precision.

These results are therefore inconsistent with Plaisier and Smeets’ (2017) conclusion that auditory and haptic information are integrated. One contributing factor explaining the difference in results we have already mentioned in the Introduction. The Plaisier and Smeets study featured a small group of participants (n = 7), which likely introduced a large measure of sampling variability. Moreover, estimates of precision were based on the variability between participants as opposed to being based on more instructive within-participant measures. One other difference that may be of interest to point out is the maximum number of objects in the container. Whereas in our study there could be up to eight objects, in the Plaisier and Smeets study there was a maximum of five. This small difference may nevertheless have significant consequences since humans have separate cognitive mechanisms for processing small and large numbers (Feigenson et al. 2004; Revkin et al. 2008). Subitizing processes small numerosities, up to about 4, rapidly and essentially without error. The Approximate Number System (ANS) processes numbers larger than that, using estimation to make approximate and imprecise judgments of numerical magnitudes (Dehaene 1997; Feigenson et al. 2004). In short, it is possible that the enumeration task in our study was in part more difficult because of the presumed involvement of the less precise ANS from about 4 items onwards (Frissen and Chen 2024; see also Fig. 2).

### Little evidence for winner-take-all favoring auditory cues

We also did not find convincing evidence for a winner-take-all mechanism that favors auditory over haptic cues as proposed by Pittinger and colleagues 1997; Pittinger and Mincy 1999. For instance, at an individual level, measures of accuracy in the auditory-haptic condition did not tend to match with the auditory-only condition; nor, for that matter, did it tend to match with the haptic-only condition. In fact, there did not seem to be a winning cue as such.

If anything, the results suggest that the winning cue, or rather the more precise cue, is individually determined, with only roughly half of the participants favoring the auditory cue and the other half favoring the haptic cue. We are currently not able to know the reason for an individual’s leaning toward either the auditory or haptic sensory modality. One explanation comes from the modality appropriateness hypothesis (Welch 1999), which holds that the modality that is the “more appropriate” for the task will dominate. For instance, while both the auditory and visual sense can provide spatial information, the latter sense is known to be orders of magnitude more precise than the former and would therefore dominate in a spatial localization task. Contrary to this localization example, there is no apparent reason to assume that either auditory or haptic sense would be more appropriate in the current container enumeration task, which might explain why we found that either sense is favored more or less equally across participants. We note that the modality appropriateness hypothesis implies that the presumed dominant modality is fixed. However, such fixedness in modality dominance might not exists and it is rather the *reliability* of the perceptual estimate of a stimulus that is instrumental (e.g., Ernst and Bülthoff 2004). Thus, any apparent dominance of modality A over modality B might be reversable by experimentally adding noise to the stimulus in modality A, and vice versa. We return to this notion in the Conclusion.

### Effect of box exploration constraints

Our secondary objective was to explore the potential effects of how participants manipulate the box on their ability to enumerate its content. The comparison across the three experiments appears to show some such procedural effects. Letting go of the time constraint (Experiment 2) did not have a noticeable effect on the results. However, letting go of both the time and movement constraints (Experiment 3) produced two effects. First, Experiment 3 was the only case in which there was some suggestion of integration. That is, for the majority of participants, the auditory-haptic condition tended to show evidence for improved accuracy and precision, although only the former was statistically significant.

Second, performance in the haptic-only condition improved considerably. Indeed, the differences in estimates between the auditory-only and haptic-only conditions, so obvious in Experiments 1 and 2, disappeared almost entirely. These findings are consistent with seminal studies investigating the role of active touch and time constraints on form recognition (Gibson 1962; Heller 1984). They are also reminiscent of studies on rod length perception (Burton and Turvey 1990; Chan 1996; Park and Rie 2014). For instance, Park and Rie (2014) investigated participants’ ability to estimate the length of a rod under various movement conditions. In one of these conditions (“self-determined”) participants moved a rod from one end while the experiment held it on the other. In another condition (“other-determined”) the situation was reversed. While in the other-determined condition participants showed sensitivity to rod length they underestimated it by about 80%. In the self-determined condition underestimation was dramatically reduced to 35%. Overall, the results reiterate the importance of letting the haptic system operate on its own terms with no artificial constraints (Klatzky et al. 1985).

### Enumeration strategies

Container studies thus far have not explicitly tried to work out which strategies participants might adopt when enumerating content. The exception is Frissen and Chen (2024), who in a study using containers with large numbers of objects (between several dozen and several hundreds), debriefed participants about what they thought was their strategy. This particular case revealed three strategies, “positive space” (participants made reference to the container’s contents when constructing their quantity judgments), “negative space” (participants make reference to the excess space in the container), and “weight” (participant explicitly mentioned using the perceived weight). Incidentally, formal investigations of the effect of weight (in studies using small numbers of objects) showed only marginal importance of weight (Frissen et al. 2023; 2025).

For the present study, however, we can only speculate as to which strategies participants used in enumerating the contents of the containers, which we assume here could be perceptual and/or take place at higher-level cognitive levels. For instance, attention may have played a role. If attention is attracted to the one of the modalities just preceding a trial, that modality will be more important in the final estimation of that trial (e.g., because of keyboard sounds or touching of the hands preceding the trial). Attention mediated cue selection has been shown extensively in visual perception (e.g. Lee et al. 1999). And in multi-sensory perception it has been shown that attention can be drawn to separate modalities (for a review see Spence 2002). A higher-level strategy might be parallel item-by-item estimation, in which case some items may have been perceived auditorily and other items haptically to be added up at a higher cognitive level. However, this would more likely yield overestimation of numbers as opposed to the underestimation that we found here and in other studies.

### Conclusion

The present work is one of only handful of studies looking at how inferences about the content of a container are affected by having concurrent access to auditory and haptic information. However, it is the first to present a comprehensive analysis, using multiple dependent variables at the level of individual participants. The results do not appear to be in line with previously proposed interpretations which suggested that auditory and haptic information would be either subject to a winner-take-all type of mechanism that favors auditory information or would be integrated. At best we found some partial and tentative evidence for an integrative mechanism when participants were released from both time and movement constraints on how to explore the container. The results therefore also bring into focus, for the first time, the potential role of the specific way people are allowed to interact with the container. Given the tentative evidence and the potential effect of how containers are explored, future work might want to readdress the integration hypothesis. One approach would be to allow for unconstrained container exploration while independently manipulating the reliability of the auditory and haptic information and assess whether this biases the number estimates in the direction of the more reliable cue.

## Data Availability

The data is published open access and freely retrievable at 10.24416/UU01-O29WTP.

## Footnotes

1 A second-order polynomial fit was warranted by the now repeatedly documented effect that number estimation tends to be increasingly underestimated as the number of objects in the container increases.

## References

[CR1] Bakeman R (2005) Recommended effect size statistics for repeated measures designs. Behav Res Methods 37:379–384. 10.3758/BF03192707 16405133 10.3758/bf03192707

[CR2] Bevan A (2014) Mediterranean containerization. Curr Anthropol 55:387–418. 10.1086/677034

[CR3] Burton G, Turvey MT (1990) Perceiving the lengths of rods that are held but not wielded. Ecol Psychol 2:295–324. 10.1207/s15326969eco0204_1

[CR4] Cabe PA, Pittenger JB (2000) Human sensitivity to acoustic information from vessel filling. J Exp Psychol Hum Percept Perform 26:313–324. 10.1037/0096-1523.26.1.313 10696620 10.1037//0096-1523.26.1.313

[CR5] Chan T-C (1996) The situational effects on haptic perception of rod length. Percept Psychophys 58:1110–1123. 10.3758/BF03206837 8920846 10.3758/bf03206837

[CR6] Dehaene S (1997) The number sense. Oxford University Press

[CR7] Ernst MO, Bülthoff HH (2004) Merging the senses into a robust percept. Trends Cogn Sci 8:162–169. 10.1016/j.tics.2004.02.002 15050512 10.1016/j.tics.2004.02.002

[CR8] Faisal AA, Selen LPJ, Wolpert DM (2008) Noise in the nervous system. Nat Rev Neurosci 9:292–303. 10.1038/NRN2258 18319728 10.1038/nrn2258 PMC2631351

[CR9] Feigenson L, Dehaene S, Spelke E (2004) Core systems of number. Trends Cogn Sci 8:307–314. 10.1016/j.tics.2004.05.002 15242690 10.1016/j.tics.2004.05.002

[CR10] Frissen I, Chen AN (2024) Humans can sense large numbers of objects in a box by touch alone. Perception 53:17–30. 10.1177/03010066231207324 37859336 10.1177/03010066231207324 PMC10798026

[CR11] Frissen I, Yao H-Y, Guastavino C, Hayward V (2022) Humans sense by touch the location of objects that roll in handheld containers. Q J Exp Psychol 76:381–390. 10.1177/17470218221086458 10.1177/17470218221086458 PMC9896261 35212251

[CR12] Frissen I, Kappassov Z, Huang KY, Ziat M (2023) Humans can sense small numbers of objects in a box by touch alone. Perception 52:799–811. 10.1177/03010066231201960 37728156 10.1177/03010066231201960 PMC10634214

[CR13] Frissen I, Xiao S, Kabdyshev N, Zabirova M, Ziat M (2025) The haptic cues humans use to sense small numbers of objects in a box. Atten Percept Psychophys 87:577–587. 10.3758/s13414-025-03011-y 39904934 10.3758/s13414-025-03011-y

[CR14] Gibson JJ (1962) Observations on active touch. Psychol Rev 69(6):477–491. 10.1037/h0046962 13947730 10.1037/h0046962

[CR15] Goodenough WH (2003) In pursuit of culture. Annu Rev Anthropol 32:1–12. 10.1146/annurev.anthro.32.061002.093257

[CR16] Haukoos JS, Lewis RJ (2005) Advanced statistics: bootstrapping confidence intervals for statistics with “difficult” distributions. Acad Emerg Med 12(4):360–365. 10.1197/j.aem.2004.11.018 15805329 10.1197/j.aem.2004.11.018

[CR17] Heller MA (1984) Active and passive touch: the influence of exploration time on form recognition. J Gen Psychol 110(2):243–249. 10.1080/00221309.1984.9709968 6726202 10.1080/00221309.1984.9709968

[CR18] Hummel, E., Pacchierotti, C., Gouranton, V., Gaugne, R., Nicolas, T., and Lécuyer, A.: Haptic rattle: Multi-modal rendering of virtual objects Inside a hollow container. In: H. Seifi, et al. (eds.). Haptics: Science, technology, applications (Lecture Notes in Computer Science, Vol. 13235). Proceedings of the 12th International EuroHaptics Conference. Springer. (2022). 10.1007/978-3-031-06249-0_22

[CR19] Jansson G (1993) Perception of the amount of fluid in a vessel shaken by a hand. In: Valenti SS, Pittenger JB (eds) Studies in Perception and Action II. Psychology Press, pp 263–267

[CR20] Jansson G, Juslin P, Poom L (2006) Liquid-specific stimulus properties can be used for haptic perception of the amount of liquid in a vessel put in motion. Perception 35:1421–1432. 10.1068/p3455 17214385 10.1068/p3455

[CR21] JASP Team.: JASP (Version 0.16.4) [Computer software]. (2023)

[CR22] Klatzky RL, Lederman SJ, Metzger VA (1985) Identifying objects by touch: an “expert system.” Percept Psychophys 37(4):299–302. 10.3758/BF03211351 4034346 10.3758/bf03211351

[CR23] Klose, A.: The container principle: how a box changes the way we think. MIT Press. (2015)

[CR24] Koshiyama R., Kikuchi T., Morita J., and Sugimoto M.: VolRec: haptic display of virtual innervolume in consideration of angular moment. In: A. D. Cheok (Ed.), Proceedings of the 12th International Conference on Advances in Computer Entertainment Technology (Article 32). Association for Computing Machinery. (2015) 10.1145/2832932.2832970

[CR25] Lee DK, Itti L, Koch C, Braun J (1999) Attention activates winner-take-all competition among visual filters. Nat Neurosci 2:375–381. 10.1038/7286 10204546 10.1038/7286

[CR26] The MathWorks, Inc.: MATLAB (Version 9.13.0, R2022b) [Computer software]. The MathWorks, Inc. (2022) https://www.mathworks.com

[CR27] Park I-J, Rie J-I (2014) Haptic perception of rod length when exploration is self-determined, other-determined, and self-other co-determined. Percept Mot Skills 119:564–575. 10.2466/24.PMS.119c25z5 25349893 10.2466/24.PMS.119c25z5

[CR28] Peirce J, Gray JR, Simpson S, MacAskill M, Höchenberger R, Sogo H, Kastman E, Lindeløv JK (2019) PsychoPy2: experiments in behavior made easy. Behav Res Methods 51:195–203. 10.3758/s13428-018-01193-y 30734206 10.3758/s13428-018-01193-y PMC6420413

[CR29] Pittenger, J. B., Jordan, J., Belden, A., Goodspeed, P., and Brown, F.: Auditory and haptic information support perception of size. In: M.A., Schmuckler, & J.M. Kennedy (Eds.), Studies in Perception and Action IV (pp. 103–105). Lawrence Erlbaum. (1997)

[CR30] Pittenger JB, Mincy MD (1999) Haptic and auditory information support perception of size: Fine granules. In: Greaty MA, Thomson JA (eds) Studies in Perception and Action V. Lawrence Erlbaum, pp 68–72

[CR31] Plaisier M. A., and Smeets J. B. J.: How many objects are inside this box? Proceedings of 2017 IEEE World Haptics Conference. June (pp. 5–9), Münich, Germany. (2017) 10.1109/whc.2017.7989908

[CR32] Revkin SK, Piazza M, Izard V, Cohen L, Dehaene S (2008) Does subitizing reflect numerical estimation? Psychol Sci 19:607–614. 10.1111/j.1467-9280.2008.02130.x 18578852 10.1111/j.1467-9280.2008.02130.x

[CR33] Scheller M, Nardini M (2024) Correctly establishing evidence for cue combination via gains in sensory precision: why the choice of comparator matters. Behav Res Methods 56:2842–2858. 10.3758/s13428-023-02227-w 37730934 10.3758/s13428-023-02227-w PMC11133123

[CR34] Sekiguchi Y., Hirota K., and Hirose M.: The design and implementation of ubiquitous haptic device [Paper presentation]. In: First Joint Eurohaptics Conference and Symposium on Haptic Interfaces for Virtual Environment and Teleoperator Systems. (2005) 10.1109/whc.2005.128

[CR35] Spence C (2002) Multisensory attention and tactile information-processing. Behav Brain Res 135:57–64. 10.1016/S0166-4328(02)00155-9 12356434 10.1016/s0166-4328(02)00155-9

[CR36] Stein, B. E. (Ed.).: The New Handbook of Multisensory Processing. MIT Press. (2012)

[CR37] Suddendorf T, Kirkland K, Bulley A, Redshaw J, Langley MC (2020) It’s in the bag: mobile containers in human evolution and child development. Evol Hum Sci 2:e48. 10.1017/ehs.2020.47 37588341 10.1017/ehs.2020.47 PMC10427442

[CR38] Suddendorf, T., and Langely, M.: Got your bag? The critical place of mobile containers in human evolution. The Conversation. (2020) https://theconversation.com/got-your-bag-the-critical-place-of-mobile-containers-in-human-evolution-142712

[CR39] Velasco C, Jones R, King S, Spence C (2013) The sound of temperature: what information do pouring sounds convey concerning the temperature of a beverage. J Sens Stud 28:335–345. 10.1111/joss.12052

[CR40] Welch, R. B.: Meaning, attention, and the “unity assumption” in the intersensory bias of spatial and temporal perceptions. In, G. Aschersleben, T. Bachman., & J. Müsseler (eds.), Advances in Psychology (Vol. 129, pp. 371–387). North-Holland. (1999) 10.1016/S0166-4115(99)80036-3

[CR41] Yao H.-Y., and Hayward V.: An experiment on length perception with a rolling stone. In: Proceedings of the 1st EuroHaptics Conference, July 3–6, Paris, pp. 325–330. (2006)

